# The Age-Friendly University: Theory and Practice in Institutional Transformation

**DOI:** 10.1093/geroni/igaa057.027

**Published:** 2020-12-16

**Authors:** Phillip Clark, Skye Leedahl

**Affiliations:** University of Rhode Island, Kingston, Rhode Island, United States

## Abstract

Growth in the network of Age-Friendly Universities (AFU) signals the importance of making higher education more age inclusive. Commitment to AFU principles creates opportunities for universities to develop new initiatives and activities that embody them. However, changing academic culture can present unique challenges as well as opportunities. Theories of organizational change help in developing strategies promoting greater involvement of older adults in communities traditionally focused on younger adults. The University of Rhode Island’s experience in continuing to develop as an AFU illustrates the complex challenge of transformation in institutions typically slow to change. This presentation focuses on the following three elements. First, uncovering and confronting ageism in academic settings is a critical first step in opening up the campus community to students of all ages. Second, identifying champions who can advocate for change in different institutional segments is an essential element in expanding the AFU movement. Third, promoting intergenerational programs helps to build bridges between traditional age and older students, particularly emphasizing the contributions that older adults can make to the academic enterprise. Implications for expanding the AFU network while focusing on individual institutions include the following: (1) using theory to drive practice in an intentional and strategic fashion; (2) identifying factors opposing change, particularly since they are usually hidden and not widely recognized; (3) developing a strategy to address these barriers, especially one tailored to the unique institutional context; and (4) recognizing the larger social, economic, and political forces generally in higher education that establish the context for AFUs.

